# Construction and application of connection number and projection pursuit models for groundwater quality evaluation: A case study of the main urban area of Bozhou City

**DOI:** 10.1371/journal.pone.0356475

**Published:** 2026-08-27

**Authors:** Zheng Li, Shangming Jiang, Juliang Jin, Rui Shen, Hongwei Yuan

**Affiliations:** 1 Water Resources Research Institute of Anhui Province and Huaihe River Commission, Ministry of Water Resources, Hefei, China; 2 Anhui Provincial Key Laboratory of Water Science and Intelligent Water Conservancy, Hefei, China; 3 School of Civil Engineering, Hefei University of Technology, Hefei, China; Ardakan University, IRAN, ISLAMIC REPUBLIC OF

## Abstract

To address the limitations of existing groundwater quality evaluation methods, such as subjective weighting, ambiguous classification boundaries, and the lack of multi-perspective robustness verification, this study constructed two quantitative groundwater evaluation frameworks by coupling the Projection Pursuit (PP) model with the Set Pair Analysis (SPA) model. First, a PP-based evaluation model was established. An Accelerated Genetic Algorithm (AGA) was employed to optimize both the projection direction and the parameters of the Logistic curve, thereby fully exploiting the intrinsic structural features of high-dimensional water quality data to achieve objective dimensionality reduction and continuous grade quantification driven by multi-index data. Second, to overcome the ambiguity at the classification boundaries of water quality grades and to validate the evaluation results, the SPA model was further introduced for fuzzy evaluation. Innovatively, the optimal projection direction derived from the PP model was utilized as the objective weight in the SPA model to quantify the degree of membership of the samples to different water quality grades. Taking the groundwater in the main urban area of Bozhou City, Anhui Province, as the study subject, the results indicated that the overall groundwater quality in this area belongs to Grade II (Class II water). The characteristic values of the water quality grades calculated by the PP model ranged from 1.89 to 2.00, while those calculated by the SPA model ranged from 1.95 to 2.03. The evaluation results of these two models, which are based on different mathematical mechanisms, exhibited high consistency. Furthermore, index perturbation sensitivity analysis and independent weight control experiments systematically verified the robustness of the evaluation results and the scientific validity of the weighting method. This study provides a rigorous theoretical basis and technical support for the precise evaluation and sustainable management of regional groundwater.

## 1. Introduction

Groundwater, as an important water resource, is widely used in different domains such as industry, agriculture, and residential life. [1,2]. With the accelerating pace of industrialization and urbanization, groundwater quality is facing increasingly severe pollution pressures [3]. Groundwater quality issues are not only related to regional ecological and environmental protection but also pose a direct threat to public health and safety, thereby constraining sustainable socio-economic development [4–6]. Therefore, conducting groundwater quality evaluation research and accurately grasping the current status of groundwater quality have become critical research directions in the fields of water resource evaluation technology development, water resource management, and ecological environmental protection [7,8].

Currently, a variety of groundwater quality evaluation models and technical methods have been developed both domestically and internationally [9–12]. Commonly used evaluation methods include the single-factor index method [13,14], the Nemerow index method [15,16], the Set Pair Analysis (SPA) connection number method [17,18], the grey relational 13,14 analysis method [19,20], and mathematical statistics analysis [21–23]. Although the comprehensive water quality index method, represented by the GWQI, is widely applied [24,25], it generally suffers from result deviations caused by subjective weighting, weak scientific validity, and poor physical interpretability. Furthermore, due to the lack of strict upper bounds for water quality classification criteria and large spans in classification intervals, this method can only achieve rough qualitative grading, making it difficult to distinguish subtle differences in water quality within the same grade. Most studies [26,27] rely solely on internal accuracy metrics (such as 𝑅^2^ and RMSE) of a single model for validation, lacking multi-perspective cross-validation based on models with different mathematical mechanisms. Consequently, the robustness and universality of the evaluation conclusions remain questionable, failing to truly reflect the objective status of regional water quality. Additionally, quantitative groundwater quality evaluation technologies with strong applicability still need to be further expanded and refined.

Therefore, how to objectively assign weights to indices, resolve the ambiguity near critical threshold values, and construct a highly applicable quantitative evaluation model that can be mutually verified from different perspectives remain urgent challenges to be overcome. As a powerful tool for mining the intrinsic structure of high-dimensional data, the Projection Pursuit (PP) method uses projection directions to represent the contribution degree of each evaluation index to the comprehensive characteristics of the system [28,29], making it a viable approach for objective weighting. Meanwhile, the SPA method overcomes the ambiguity near critical threshold values by quantifying the membership tendency of samples to different water quality grades, explaining the attribution mechanism of water quality grades from the perspective of identity-discrepancy-contrary data structures. Since the PP and SPA models are based on different mathematical mechanisms, if their evaluation results exhibit high consistency, it will strongly corroborate the robustness and objectivity of the evaluation conclusions.

To address the aforementioned shortcomings, this study first constructed a comprehensive evaluation index system for regional groundwater quality. Based on the water quality grade thresholds determined by national standards, random simulation technology was utilized to generate 150 sets of standard training samples covering the entire feature space. On this basis, an Accelerated Genetic Algorithm (AGA) was employed to optimize and solve for the optimal projection direction and Logistic curve parameters, establishing a nonlinear fitting relationship between the comprehensive projection values and water quality grades. This approach fully exploited the intrinsic features of high-dimensional water quality monitoring data, thereby constructing a groundwater quality evaluation model based on PP. To overcome the defects of subjective weighting, this study innovatively utilized the optimal projection direction derived from the PP model as the objective weight for each evaluation index. The degree of membership of a single index belonging to different water quality grades was quantified and weighted to calculate the comprehensive connection number. Combined with the grade characteristic value method to determine the water quality grade, an evaluation model based on the SPA connection number was constructed. Taking the groundwater in the main urban area of Bozhou City, Anhui Province, as the research subject for empirical evaluation, the evaluation results from the two models with different mathematical mechanisms were cross-validated to corroborate the rationality of the constructed models and the scientific validity of the weighting method. Furthermore, index perturbation sensitivity analysis and independent weight control experiments were supplemented to systematically verify the stability of the weighting system and the independence of the models. This study aims to refine the multi-index quantitative evaluation technical system for groundwater, providing a rigorous theoretical basis and technical support for the precise assessment of regional groundwater quality status, groundwater resource security control, and sustainable development and utilization.

## 2. Materials and methods

### 2.1. Study area

**Fig 1:**
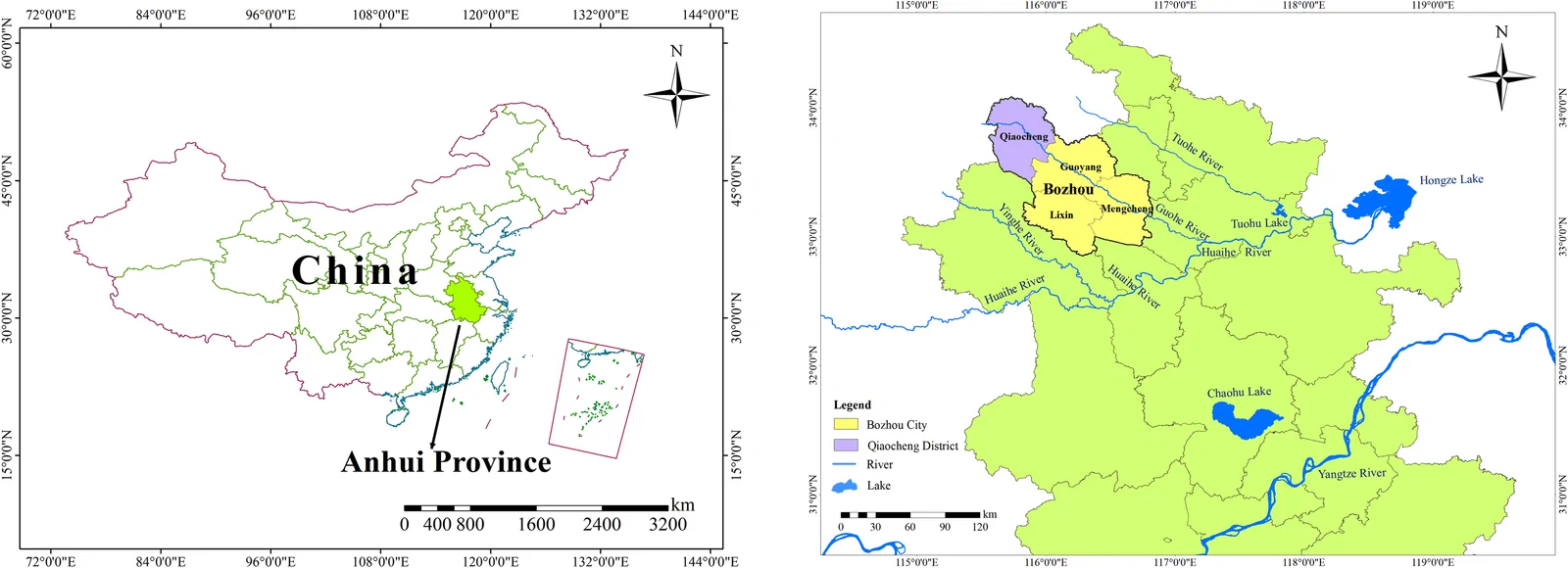
Location of the study area. (The map is available at the following link: http://bzdt.ch.mnr.gov.cn/index.html).

Bozhou is located in the northwestern part of Anhui Province, at the southern end of the North China Plain, where the borders of Anhui and Henan Province meet. Bozhou City suffers from severe water scarcity, with per capita water resources of only 346 m^3^, far below the internationally recognized safety threshold of 1,000 m^3^, making it a severely water-scarce region. Qiaocheng District is the main urban area of Bozhou City, located in its northwestern part. The location of the study area is shown in Fig 1.

According to the 2022 Anhui Province Water Resources Bulletin and the Bozhou City Water Resources Bulletin, the total water supply in Bozhou City was 11.08 × 10⁸ m^3^, of which groundwater accounted for 5.12 × 10⁸ m^3^, representing nearly 50% of the total. Meanwhile, the percentage of surface water and groundwater in the water supply was 41.1% and 49.4% in Qiaocheng District, respectively. This highlights the importance of conducting a reasonable evaluation of groundwater quality in Bozhou City for the purpose of scientifically managing groundwater resources. The regional groundwater quality evaluation model based on projection pursuit and the regional groundwater quality evaluation model based on connection number were applied to the evaluation of groundwater quality in Bozhou City.

### 2.2. Data sources

The water quality data used in our study were obtained from the reference [30] (DOI:10.3969/j.issn.1008-9500.2023.08.018). According to the “Groundwater Quality Standards” (GB/T 14848–2017), 15 indices were selected to evaluate the groundwater quality. The groundwater monitoring data are presented in the Table 1.

**Table 1:** Monitoring data of groundwater in Bozhou City.

Target level	Indices level	DXS01	DXS02	DXS03	DXS04
Evaluation of Groundwater Quality in Bozhou City	*x*_1_, Hg/(mg/L)	0.00004	0.00004	0.00004	0.00004
	*x*_2_, Cd/(mg/L)	0.0001	0.0001	0.0001	0.0002
	*x*_3_, As/(mg/L)	0.0036	0.0022	0.0051	0.003
	*x*_4_, Pb/(mg/L)	0.002	0.002	0.002	0.002
	*x*_5_, Cr^6 +^ /(mg/L)	0.004	0.004	0.004	0.004
	*x*_6_，Mn/(mg/L)	0.01	0.02	0.02	0.04
	*x*_7_, NH^4 +^ -N/(mg/L)	0.05	0.053	0.058	0.057
	*x*_8_, NO^2-^-N/(mg/L)	0.003	0.071	0.016	0.017
	*x*_9_, F^-^/(mg/L)	1.2	1.143	1.29	1.47
	*x*_10_, CODMn/(mg/L)	1	0.8	0.8	1
	*x*_11_, NO^3-^-N/(mg/L)	0.17	2.5	0.66	1.15
	*x*_12_, SO4^2-^/(mg/L)	201	154	171	203
	*x*_13_, Cl^-^/(mg/L)	240	181	131	104
	*x*_14_, Total hardness/(mg/L)	120	199	182	168
	*x*_15_, TDS/(mg/L)	982	878	786	857

### 2.3. Methods

**Fig 2:**
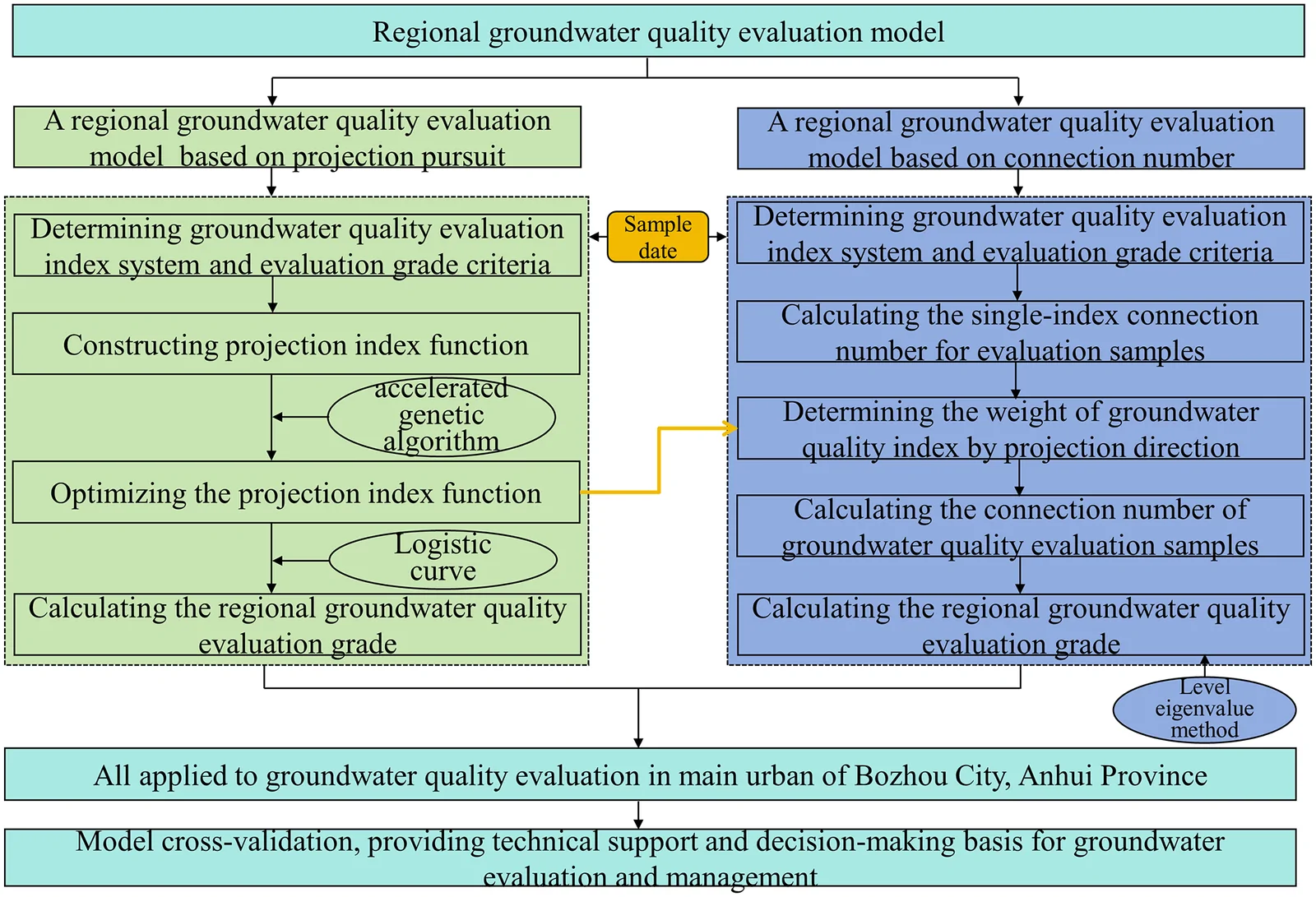
Construction of regional groundwater quality evaluation models.

To address the credibility issues caused by algorithmic limitations in single evaluation models, we have developed an evaluation framework based on different mathematical mechanisms. The Projection Pursuit (PP) model is specifically designed for dimensionality reduction and structure mining of high-dimensional data. It searches for the optimal projection direction of the data from a global perspective to establish a mapping relationship between water quality grades and projection values. Meanwhile, the Set Pair Analysis (SPA) model focuses on quantifying the uncertainty of similarity, difference, and opposition within a system. Starting from the sample values of individual indices and their membership degrees to evaluation grades, it constructs an identity-discrepancy-contrary connection number structure for each individual index. By assigning objective weights to these indices, a comprehensive connection number is obtained, thereby establishing the relationship between water quality grades and the components of the connection number. A regional groundwater quality evaluation model based on projection pursuit, including 4 steps, and, a regional groundwater quality evaluation model based on connection number, including 5 steps, shown in Fig 2.

#### 2.3.1. Projection pursuit evaluation model.

**Step 1**: Establish the regional groundwater quality evaluation indices system and evaluation grade criteria. Based on the “Groundwater Quality Standards” (GB/T 14848–2017), comprehensively consider the availability, representativeness, and scientific validity of regional groundwater evaluation indices to construct the regional groundwater quality evaluation indices system {*x*_*j*_|*j* = 1, 2, …, *n*_*j*_}, with the corresponding evaluation indices dataset being {x*(*i*, *j*)| *i* = 1,2, …, *n*_*i*_, *j* = 1, 2, …, *n*_*j*_}, where *n*_*i*_ and *n*_*j*_ represent the number of samples and the number of evaluation indices, respectively. The number of evaluation grades and the evaluation grade criteria are derived from the above mentioned standard, where evaluation grades are divided into 1, 2, 3, 4, and 5, representing Class I, Class II, Class III, Class IV, and Class V, respectively [30–32].

**Step 2:** Construct the projection index function. Based on the groundwater quality evaluation standards specified in the “Groundwater Quality Standards” (GB/T 14848–2017), randomly generate 30 samples each for the 5 criteria grades (1, 2, 3, 4, and 5), totaling 150 samples {x*(*i*, *j*)| *i* = 1,2, …, 150, *j* = 1, 2, …, 15}, with the corresponding empirical criteria grades being y(*i*). For example, if the *n*_*j*_ evaluation indices of the 30 samples meet the requirements of grade 1, the corresponding criteria grade is 1. The purpose of constructing a groundwater quality evaluation model based on projection pursuit is to establish a quantitative relationship between x*(*i*, *j*) and *y*(*i*). The projection pursuit method can achieve dimensionality reduction projection of the *n*_*j*_-dimensional data {x*(*i*, *j*)|*j* = 1, 2, …, *n*_*j*_} using ***a***=(*a*(1),*a*(2), …,*a*(*n*_*j*_)) as the projection direction to obtain a one-dimensional projection value *z*(*i*) [33–35] (Eq. 1):


$$\begin{array}{c} z\left(i\right)=\sum_{j=1}^{n_{j}}a\left(j\right)x\left(i,j\right), \end{array}$$ (1)

where ***a*** is the unit length vector. The projection value *z(i)* obtained from Formula (1) can be mathematically related to the empirical criteria grade *y*(*i*).

To make the projection pursuit method generalizable, {x*(*i*, *j*)| *j* = 1, 2, …, *n*_*j*_} is normalized to {*x*(*i*, *j*)|*j* = 1, 2, …, *n*_*j*_} [33,34,36] (Eq. 2):


$$\begin{array}{c} x\left(i,j\right)=x^{*}\left(i,j\right)/x_{\text{max}}\left(j\right), \end{array}$$ (2)

where *x*_max_(*j*) is the maximum value of the *j*-th evaluation index {x*(*i*,*j*)|*i* = 1 ~ *n*_*i*_} for groundwater quality evaluation.

The core component of the projection pursuit method is how to construct a projection index function to identify the optimal projection direction that can reveal the complex data structure of groundwater quality evaluation. Two main aspects are considered here: first, the standard deviation of the one-dimensional projection value *z*(*i*) should be as large as possible to reflect as much variability information as possible from {*x*(*i*,*j*)}; second, the absolute value of the correlation coefficient |*R*_*zy*_| between the one-dimensional projection value *z*(*i*) and empirical criteria grades *y*(*i*) should be as large as possible to ensure good synchronization and interpretability between the projection values and empirical criteria grades. For this purpose, the projection index function can be constructed as [33,34,37] (Eq. 3):


$$\begin{array}{c} Q\left(a\right)=S_{z}\left|R_{zy}\right|, \end{array}$$ (3)

where | | is the absolute value, and *S*_*z*_ is the standard deviation of the projection value *z*(*i*), [33,34,38] (Eq. 4):


$$\begin{array}{c} S_{z}=[\sum_{i=1}^{n_{i}}(z(i)-Ez{)}^{\text{2}}/(n_{i}-1){]}^{0.5}, \end{array}$$ (4)

*R*_*zy*_ is the correlation coefficient between *z*(*i*) and *y*(*i*) [38] (Eq. 5):


$$\begin{array}{c} R_{zy}=\frac{\sum_{i=1}^{n_{i}}(z(i)-Ez)(y(i)-Ey)}{[\sum_{i=1}^{n_{i}}(z(i)-Ez{)}^{\text{2}}\sum_{i=1}^{n_{i}}(y(i)-Ey{)}^{\text{2}}{]}^{0.5}}, \end{array}$$ (5)

where *Ez* and *Ey* are the means of {*z*(*i*)|*i* = 1, 2, …, *n*_*i*_} and {*y*(*i*)|*i* = 1, 2, …, *n*_*i*_}, respectively.

**Step 3**: Optimize the projection index function. Given the empirical criteria level *y*(*i*) and the sample data of the evaluation index {x*(*i*, *j*)| *i* = 1, 2, …, *n*_*i*_, *j* = 1, 2, …, *n*_*j*_}, the projection index function *Q*(***a***) is only a function of the projection direction **a**. Different projection directions reflect different data structure characteristics. The optimal projection direction is the one that maximally exposes certain feature structures of high-dimensional data. The absolute values of the components of the optimal projection direction essentially reflect the influence of each water quality index on the water quality grade. The optimal projection direction can be determined by solving the problem of maximizing the projection index function [37] (Eqs.6 and 7), i.e.,


$$\begin{array}{c} max\;Q\left(a\right)=S_{z}\left|R_{zy}\right|, \end{array}$$ (6)

$$\begin{array}{c} s.t.\text{\textbackslash{}hspace\{0.17em}\;\;\}\sum_{j=1}^{n_{j}}a^{\text{2}}\left(j\right)=1. \end{array}$$ (7)

This is clearly a complex nonlinear optimization problem with {*a*(*j*)| *j* = 1, 2, …, *n*_*j*_} *n*_*j*_ optimization variables, which can be easily solved using an AGA [39–41]. The AGA is a general and universal optimization method.

**Step 4**: Calculate the regional groundwater quality evaluation grade. Substitute the optimal projection direction a* obtained in Step 3 into Eq.1 to obtain the calculated value z*(*i*) of the *i*-th sample projection value. Construct a function expression based on the scatter plot of z*(*i*) and the empirical standard grade *y*(*i*). Relevant studies have shown that the Logistic curve is a very suitable model for grade evaluation [39,42] (Eq. 8), namely:


$$\begin{array}{c} y^{*}\;\left(i\right)=\frac{N}{1+e^{c_{\text{1}}-c_{\text{2}}z*\left(i\right)}}, \end{array}$$ (8)

where y*(*i*) is the calculated value for the *i*-th sample grade; *N* is the maximum water quality grade, taken as 5 in this paper; *c*_1_ and *c*_2_ are undetermined parameters, which can be optimized using an accelerated genetic algorithm to determine the following objective function [39,41] (Eq. 9):


$$\begin{array}{c} \min\;F(c_{1},c_{2})=\sum_{i=1}^{n_{i}}(y^{*}(i)-y{\left(i)\right)}^{2} \end{array}$$ (9)

It can be seen that once the parameters *c*_1_, *c*_2_, and the optimal projection direction a* are determined, they can be substituted into Eqs. (1), (2), and (8) to determine the groundwater quality grade to be evaluated. The AGA performs global optimization for the PP model, mining the “optimal projection direction” that maximally reflects the differences in water quality grades and the intrinsic data structure. Subsequently, the Logistic curve is used to construct a continuous mapping relationship between the projection values and the water quality grades. This enables the output of refined, continuous grade values, thereby overcoming the limitations of discrete integer grading based on fixed threshold intervals.

All mathematical calculations and algorithm optimization were implemented via MATLAB R2016a. The self-programmed codes for accelerated genetic algorithm, projection pursuit were written in native MATLAB script without third-party toolboxes. Specifically, the genetic algorithm employed is the Real-coded Accelerating Genetic Algorithm (RAGA). The population size was set to 300, with the number of superior individuals set to 5. The RAGA iteration process was terminated after 100 accelerating cycles. For the genetic operators, the Roulette Wheel Selection method was used for the selection operation. The crossover operation generated offspring through random linear combinations, and the mutation probability was determined based on the selection probability. Basic statistical sorting of monitoring data was completed using Microsoft Excel 2016.

#### 2.3.2. Connection number evaluation model.

**Step 1**: Same as Step 1 in Section 2.3.1.

**Step 2**: Calculate the single-index connection number for the regional groundwater quality evaluation sample. Set Pair Analysis (SPA) Theory was proposed by Chinese scholar Zhao Keqin in 1989. It is based on dialectical materialism and serves as a novel uncertainty analysis tool that studies uncertainty and certainty as a unified system [17,18]. SPA requires the construction of two related sets, followed by an analysis of their similarity, difference, and opposition. The degree of connection between the two sets is quantified using a connection number, where the certainty connection between the two sets is divided into similarity connection and opposition connection, and the uncertainty connection is difference connection [43].

This paper constructs two sets: groundwater evaluation index data x*(*i*, *j*) and evaluation criteria grades. The degree of connection between the evaluation index data and the evaluation criteria grades, i.e., the single-index connection number *u*_*ij*_, can be expressed as a five-element connection number [17,18,43] (Eq. 10).


$$\begin{array}{c} u_{ij}=v_{ij1}+v_{ij2}I_{\text{1}}+v_{ij3}I_{\text{2}}+v_{ij4}I_{\text{3}}+v_{ij5}J, \end{array}$$ (10)

where *v*_*ij*1_, *v*_*ij*2_, *v*_*ij*3_, *v*_*ij*4_, *v*_*ij*5_ are the connection number component. *I*_1_、*I*_2_、*I*_3_ are the difference coefficient; *J* is the opposition coefficient.

The connection number component *u*_*ijk*_(*k* = 1,2,3,4,5) is quantitatively analyzed using the following formula [18,44] (Eqs. 11-15):


$$\begin{array}{c} \left\{ \begin{array}{c} @l@u_{ij1}=1\\ u_{ij2}=1-2\left(s_{1j}-x^{*}\left(i,j\right)\right)/(s_{1j}-s_{0j})\\ u_{ij3}=-1\\ u_{ij4}=-1\\ u_{ij5}=-1 \end{array}\right.,\text{\textbackslash{}hspace\{0.17em}\}\left(\begin{array}{c} @l@s_{0j}<x^{*}\left(i,j\right)\leq s_{1j}\text{ forpositiveindex}\text{,}\\ \text{or }s_{0j}>x^{*}\left(i,j\right)\geq s_{1j}\text{ fornegativeindex} \end{array}\right), \end{array}$$ (11)

$$\begin{array}{c} \left\{ \begin{array}{c} @l@u_{ij1}=1-2\left(x^{*}\left(i,j\right)-s_{1j}\right)/(s_{2j}-s_{1j})\\ u_{ij2}=1\\ u_{ij3}=1-2\left(s_{2j}-x^{*}\left(i,j\right)\right)/(s_{2j}-s_{1j})\\ u_{ij4}=-1\\ u_{ij5}=-1 \end{array}\right.,\text{\textbackslash{}hspace\{0.17em}\}\left(\begin{array}{c} @l@s_{1j}<x^{*}\left(i,j\right)\leq s_{2j}\text{ forpositiveindex}\text{,}\\ \text{or }s_{1j}>x^{*}\left(i,j\right)\geq s_{2j}\text{ fornegativeindex} \end{array}\right), \end{array}$$ (12)

$$\begin{array}{c} \left\{ \begin{array}{c} @l@u_{ij1}=-1\\ u_{ij2}=1-2\left(x^{*}\left(i,j\right)-s_{2j}\right)/(s_{3j}-s_{2j})\\ u_{ij3}=1\\ u_{ij4}=1-2\left(s_{3j}-x^{*}\left(i,j\right)\right)/(s_{3j}-s_{2j})\\ u_{ij5}=-1 \end{array}\right.,\left(\begin{array}{c} @l@s_{2j}<x^{*}\left(i,j\right)\leq s_{3j}\text{ forpositiveindex}\text{,}\\ \text{or }s_{2j}>x^{*}\left(i,j\right)\geq s_{3j}\text{ fornegativeindex} \end{array}\right), \end{array}$$ (13)

$$\begin{array}{c} \left\{ \begin{array}{c} @l@u_{ij1}=-1\\ u_{ij2}=-1\\ u_{ij3}=1-2\left(x^{*}\left(i,j\right)-s_{3j}\right)/(s_{4j}-s_{3j})\\ u_{ij4}=1\\ u_{ij5}=1-2\left(s_{4j}-x^{*}\left(i,j\right)\right)/(s_{4j}-s_{3j}) \end{array}\right.,\left(\begin{array}{c} @l@s_{3j}<x^{*}\left(i,j\right)\leq s_{4j}\text{ forpositiveindex}\text{,}\\ \text{or }s_{3j}>x^{*}\left(i,j\right)\geq s_{4j}\text{ fornegativeindex} \end{array}\right), \end{array}$$ (14)

$$\begin{array}{c} \left\{ \begin{array}{c} @l@u_{ij1}=-1\\ u_{ij2}=-1\\ u_{ij3}=-1\;\\ u_{ij4}=1-2(x^{*}(i,j)-s_{4j})/(s_{5j}-s_{4j})\;\\ u_{ij5}=1 \end{array}\right.,\left(\begin{array}{c} @l@s_{4j}<x^{*}\left(i,j\right)\leq s_{5j}\text{ forpositiveindex}\text{,}\\ \text{or}s_{4j}>x^{*}\left(i,j\right)\geq s_{5j}\text{ fornegativeindex} \end{array}\right), \end{array}$$ (15)

where *s*_1*j*_, *s*_2*j*_, *s*_3*j*_ and *s*_4*j*_ are the critical value of evaluation grade 1 and 2, grade 2 and 3, grade 3 and 4,and grade 4 and 5, respectively. *s*_0*j*_ and *s*_5*j*_, the other critical value of evaluation grade 1 and grade 5.

By substituting any groundwater evaluation index value into Eqs. 10–14, the degree of connection with evaluation grades 1, 2, 3, 4, and 5 can be obtained, thereby achieving quantitative analysis. However, according to SPA theory, the connection number component should be converted into relative affiliation degree, which is between 0 and 1. Therefore, a relative affiliation degree function needs to be constructed and normalized to obtain the connection number component *v*_*ijk*_ [18,45] (Eqs. 16 and 17):


$$\begin{array}{c} v_{ijk}^{*}=0.5+0.5u_{ijk}, \end{array}$$ (16)

$$\begin{array}{c} v_{ijk}=\frac{v_{ijk}^{*}}{v_{ij1}^{*}+v_{ij2}^{*}+v_{ij3}^{*}}. \end{array}$$ (17)

The single-index five-element connection number *u*_*ij*_ can be composed of the connection number components *v*_*ijk*_, from which the expression of Eq.10 can be obtained.

**Step 3**: Calculate the weights of the regional groundwater quality evaluation indices. The reasonable determination of evaluation indices is crucial to the comprehensive evaluation of the system, and how to consider the degree of influence of each evaluation index on the groundwater quality system has always been a difficult issue. According to the basic principle of projection pursuit, the absolute values of the components of the optimal projection direction essentially reflect the degree of influence of each groundwater quality index on the water quality grade. Therefore, this paper proposes to determine the weights of evaluation indices based on the optimal projection direction.

As the unit vector ***a*** for the optimal projection direction can be negative, absolute value transformation is performed on vector ***a***[46] (Eq. 18):


$$\begin{array}{c} {𝐚}^{*}=\left|𝐚\right|, \end{array}$$ (18)

clearly, the elements of vector a* are non-negative and reflect the degree of influence of groundwater quality evaluation indices on groundwater quality at this point. After normalization, the weights of each groundwater quality evaluation index can be obtained (Eq. 19):


$$\begin{array}{c} w\left(j\right)=a^{*}\left(j\right)/\sum_{j=1}^{n_{j}}a^{*}\left(j\right). \end{array}$$ (19)

The weighting calculation method based on the optimal PP projection direction provides fully data-driven index weights and comprehensively mines the intrinsic structure of high-dimensional data, demonstrating superior performance over traditional subjective and simple objective weighting methods.

**Step 4**: Calculate the comprehensive connection number *u*_*i*_ of the regional groundwater quality evaluation sample *i*. Based on the single-index connection number in Step 2 and the weight of each index in Step 3, the comprehensive connection number *u*_*i*_[10] of the sample can be obtained by weighting [47] (Eq. 20).


$$\begin{array}{c} u_{i}=v_{i1}\text{+}v_{i2}I_{\text{1}}+v_{i3}I_{\text{2}}+v_{i4}I_{\text{3}}+v_{i5}J\text{\textbackslash{} }=\;\sum_{j=1}^{n_{j}}w_{j}v_{ij1}+\sum_{j=1}^{n_{j}}w_{j}v_{ij2}I_{\text{1}}+\sum_{j=1}^{n_{j}}w_{j}v_{ij3}I_{\text{2}}+\sum_{j=1}^{n_{j}}w_{j}v_{ij4}I_{\text{3}}+\sum_{j=1}^{n_{j}}w_{j}v_{ij5}J. \end{array}$$ (20)

**Step 5**: To fully exploit the information contained in the connection number, use the level eigenvalue method to calculate the groundwater quality grade value corresponding to the connection number of the regional groundwater quality evaluation sample *i*, as the evaluation result [48] (Eq. 21):


$$\begin{array}{c} g_{i}=\sum_{k=1}^{\text{5}}v_{ik}k, \end{array}$$ (21)

where *g*_*i*_ is the regional groundwater quality evaluation grade value, and k is the value of the five evaluation grades, i.e., *k* = 1, 2, 3, 4, 5. Eqs. 11–15 and 21 demonstrate that by constructing a multi-dimensional similarity -difference- opposition connection number structure, the SPA method can quantitatively characterize the degree of membership of each water sample to different water quality grades. It accurately depicts the gradual evolution and fuzzy transition characteristics of water quality states, effectively avoiding misjudgments and discrimination deviations for samples near critical thresholds. Furthermore, it can also output refined, continuous grade values rather than discrete integer grades.

All mathematical calculations and algorithm optimization were implemented via MATLAB R2016a. The self-programmed codes for connection number were written in native MATLAB script without third-party toolboxes. Basic statistical sorting of monitoring data was completed using Microsoft Excel 2016.

#### 2.3.3. Sensitivity analysis of index weights.

To verify the robustness and physical interpretability of the index weights under the optimal projection direction of the Projection Pursuit (PP) model, and to identify the sensitivity of the weights to fluctuations in individual index data, a one-factor perturbation sensitivity analysis was conducted in this study. Specifically, index *x*_9_ in the groundwater quality evaluation system was selected as the test subject. While keeping the data of the other 14 indices unchanged, the data for *x*_9_ were scaled by applying perturbation coefficients of 0.8, 1.0, and 1.2. These three perturbed datasets were then sequentially input into the PP model to obtain the corresponding weight sequences of the optimal projection directions. By comparing the magnitude of weight variations for the same index under different perturbation coefficients, the sensitivity characteristics of the index weights to minor data fluctuations were quantitatively revealed. This analysis corroborates that the index weights derived from the optimal projection direction possess reliable robustness and clear physical significance.

#### 2.3.4. Multi-weighting method verification scheme.

In this study, the index weights determined by the optimal projection direction were incorporated into the connection number method. The comprehensive connection number for each sample was obtained by weighting the single-index connection numbers. To avoid endogeneity issues caused by overlapping data processing between the optimal projection direction weights and the connection number evaluation model, this study employed the highly versatile objective weighting method based on the Analytic Hierarchy Process and the Accelerated Genetic Algorithm [49] (AGA-FAHP) for independent validation. Specifically, the judgment matrix was constructed based on the standard deviation of the data samples, and the genetic algorithm was employed to optimize and solve the modified matrix and index weights. This approach is classified as a single-index statistical feature-driven objective weighting method. The resulting weights were applied as the control group in the Set Pair Analysis (SPA) connection number evaluation model, and the evaluation results were compared with those obtained using the original Projection Pursuit (PP) weights (which represent a data structure-driven objective weighting method). The modeling principles, optimization objectives, and solving processes of these two weighting methods are completely independent of each other.

**Step 1**: **Standardization of evaluation index data** (Eq. 22)


$$\begin{array}{c} r\left(i,j\right)=\left(x_{\text{max}}\left(i\right)-x^{*}\left(i,j\right)\right)/\left(x_{\text{max}}\left(i\right)-x_{\text{min}}\left(i\right)\right), \end{array}$$ (22)

where *x*_max_(*i*) and *x*_min_(*i*) are the maximum and minimum values of the i -th index in the regional groundwater evaluation dataset, respectively; and *r*(*i*, *j*) is the standardized value of the evaluation index.

**Step 2**: **Construction of the Judgment Matrix**

The standard deviation of the original sample sequences for the 15 groundwater evaluation indices was used to characterize the degree of data dispersion for each index. A larger standard deviation indicates a stronger discriminative power of the index samples and a higher potential contribution to the water quality evaluation results. The judgment matrix was constructed based on the ratios of the standard deviations among the indices (Eqs. 23–25):


$$\begin{array}{c} s(i)=(\sum_{j=1}^{m}(r\left(i,j\right)-\overset{―}{r_{i}}{)}^{\text{2}}/m{)}^{0.5}, \end{array}$$ (23)

$$\begin{array}{c} \overset{―}{r_{i}}=\sum_{j=1}^{m}r\left(i,j\right)/m, \end{array}$$ (24)

$$\begin{array}{c} b_{ij}=\left\{ \begin{array}{c} @l\frac{s(i)-s(j)}{s_{max}-s_{min}}(b_{m}-1)+1,\;s\left(i\right)\geq s\left(j\right)\;\\ {}[8pt]1/(\frac{s\left(i\right)-s\left(j\right)}{s_{max}-s_{min}}\left(b_{m}-1\right)+1),\;s\left(i\right)<s\left(j\right), \end{array}\right. \end{array}$$ (25)

where *s*_min_ and *s*_max_ are the minimum and maximum values of the set {*s*(*i*)｜*i* = 1, 2, …,*n*}, respectively; the relative importance parameter is defined as *b*_*m*_ = min{9, int [*s*_max_/*s*_min_ + 0.5]}, where min and int denote the minimum function and the rounding function, respectively.

**Step 3**: **Optimization using the AGA**

In the Accelerated Genetic Algorithm (AGA), the population size was set to 300, and the number of elite individuals was set to 5. The iteration was terminated after 100 RAGA acceleration cycles. For the genetic operators, the Roulette Wheel Selection method was adopted for the selection operation. The crossover operation generated offspring through random linear combinations, utilizing the rand() function to produce random numbers. Furthermore, the mutation probability was determined based on the aforementioned selection probability.

## 3. Results

### 3.1. Results of projection pursuit model

Based on the “Groundwater Quality Standards” (GB/T 14848–2017) and the actual conditions of Qiaocheng District, an evaluation indices system and evaluation grade criteria for groundwater quality in Qiaocheng District, was established, covering sensory characteristics and general chemical indices, and toxicological indices, as shown in Table 2.

**Table 2:** Groundwater quality evaluation indices and grade criteria for Qiaocheng District, Bozhou City.

Target level	Indices level	Grade 1	Grade 2	Grade 3	Grade 4	Grade 5
		(I)	(II)	(III)	(IV)	(V)
Evaluation of Groundwater Quality in Bozhou City	*x*_1,_, Hg/(mg/L)	0 ~ 0.0001	≤0.0001	≤0.001	≤0.002	0.002 ~ 0.004
	*x*_2_, Cd/(mg/L)	0 ~ 0.0001	≤0.001	≤0.005	≤0.01	0.01 ~ 0.02
	*x*_3_, As/(mg/L)	0 ~ 0.001	≤0.001	≤0.01	≤0.05	0.05 ~ 0.10
	*x*_4_, Pb/(mg/L)	0 ~ 0.005	≤0.005	≤0.01	≤0.10	0.10 ~ 0.20
	*x*_5_, Cr^6 +^ /(mg/L)	0 ~ 0.005	≤0.01	≤0.05	≤0.10	0.10 ~ 0.20
	*x*_6_, Mn/(mg/L)	0 ~ 0.05	≤0.05	≤0.10	≤1.50	1.50 ~ 3.0
	*x*_7_, NH^4 +^ -N/(mg/L)	0 ~ 0.02	≤0.10	≤0.50	≤1.50	1.50 ~ 3.0
	*x*_8_, NO^2-^-N/(mg/L)	0 ~ 0.01	≤0.10	≤1.00	≤4.80	4.80 ~ 9.60
	*x*_9_, F^-^/(mg/L)	0 ~ 1.0	≤1.0	≤1.0	≤2.0	2.0 ~ 4.0
	*x*_10_, CODMn/(mg/L)	0 ~ 1.0	≤2.0	≤3.0	≤10.0	10.0 ~ 20.0
	*x*_11_, NO^3-^-N/(mg/L)	0 ~ 2.0	≤5.0	≤20.0	≤30.0	30.0 ~ 60.0
	*x*_12_, SO4^2-^/(mg/L)	0 ~ 50	≤150	≤250	≤350	350 ~ 700
	*x*_13_, Cl^-^/(mg/L)	0 ~ 50	≤150	≤250	≤350	350 ~ 700
	*x*_14_, Total hardness/(mg/L)	0 ~ 150	≤300	≤450	≤650	650 ~ 1300
	*x*_15_, TDS/(mg/L)	0 ~ 300	≤500	≤1000	≤2000	2000 ~ 4000

A total of 150 labelled calibration samples {x*(*i*, *j*)| *i* = 1,2, …, 150, *j* = 1, 2, …, 15} were obtained by randomly generating 30 indices datasets within each grade interval specified in Table 1. This sample set was used for parameter optimization and effectiveness verification of the projection pursuit model. And all sample datasets were standardized following (Eq.2). The established projection index function (Eq.6). simultaneously captured the internal variation of sample data and the correlation between projection values and empirical classification grades, which formed a nonlinear optimization framework for subsequent parameter solving, and the relevant optimization results are summarized in Table 3.

**Table 3:** Calculate the maximum value of Eq.6 using AGA.

Acceleration times	The range of changes for outstanding individuals			Maximum objective function value
	a(1)	…	a(15)	
0	[−1,1]	[−1,1]	[−1,1]	
100	[0.448960970,0.448960983]	…	[0.429311710,0.429311726]	0.955
RAGA optimization results	0.448960974	…	0.429311721	0.955

The optimal projection direction vector for groundwater quality evaluation in Qiaocheng District was derived via (Eq. 6), expressed as a* =(0.27,0.272,0.265,0.244,0.262,0.259,0.284,0.26,0.208,0.258,0.272,0.265,0.256,0.234,0.258).

Substitution of this vector into (Eq.1) yielded the projection value z*(*i*) for each sample. A logistic fitting curve was further constructed based on the scatter relationship between the obtained projection values z*(*i*) and the corresponding empirical standard grades *y*(*i*).

The objective function defined in (Eq.9) was optimized using the AGA, yielding the optimized model parameters *c*_1_ = 1.13 and *c*_2_ = −2.16. The fitting comparison between empirical standard groundwater quality grades *y*(*i*)and the grades y*(*i*)predicted by the projection pursuit model is presented in Table 4 (partial results). The all result are shown in S1 Table. The evaluation results of the fitting performance of the projection pursuit model are shown in Table 5.

**Table 4:** Comparison of empirical values and projection pursuit model calculation values for groundwater quality grades.

Samples	Projection value	Grade	
		Empirical values	Projection pursuit model calculation values
1	0.109	1	1.45
2	0.100	1	1.43
3	0.085	1	1.39
…	…	…	…
148	2.957	5	4.97
149	2.839	5	4.97
150	2.740	5	4.96

**Table 5:** Fitting performance evaluation of the projection pursuit model.

RMSE	MAE	R2	Maximum error	Classification accuracy
0.29	0.25	0.96	0.50	1

Groundwater quality grades of the four monitoring points in Qiaocheng District were calculated by inputting the field-measured indices datasets into Eqs (1), (2) and (8), with the evaluation results listed in Table 6.

**Table 6:** Groundwater quality evaluation grades based on projection pursuit in Qiaocheng District, Bozhou City.

Samples	Sample 1	Sample 2	Sample 3	Sample 4
Projection value	0.339	0.312	0.296	0.310
Grade values	2.00	1.94	1.89	1.93

### 3.2 Results of connection number model

Single-index connection number was computed to quantify the affiliation relationship between monitoring index values and grades, as shown in Table 7.

**Table 7:** Single-index connection number of groundwater quality evaluation.

Index	Single-index connection number			
*x*_1_	[0.714,0.286,0,0,0]	[0.714,0.286,0,0,0]	[0.714,0.286,0,0,0]	[0.714,0.286,0,0,0]
*x*_2_	[0.5,0.5,0,0,0]	[0.5,0.5,0,0,0]	[0.5,0.5,0,0,0]	[0.444,0.5,0.056,0,0]
*x*_3_	[0,0.356,0.5,0.144,0]	[0,0.433,0.5,0.067,0]	[0,0.272,0.5,0.228,0]	[0,0.389,0.5,0.111,0]
*x*_4_	[0.714,0.286,0,0,0]	[0.714,0.286,0,0,0]	[0.714,0.286,0,0,0]	[0.714,0.286,0,0,0]
*x*_5_	[0.556,0.444,0,0,0]	[0.556,0.444,0,0,0]	[0.556,0.444,0,0,0]	[0.556,0.444,0,0,0]
*x*_6_	[0.833,0.167,0,0,0]	[0.714,0.286,0,0,0]	[0.714,0.286,0,0,0]	[0.556,0.444,0,0,0]
*x*_7_	[0.313,0.5,0.188,0,0]	[0.294,0.5,0.206,0,0]	[0.262,0.5,0.238,0,0]	[0.269,0.5,0.231,0,0]
*x*_8_	[0.769,0.231,0,0,0]	[0.161,0.5,0.339,0,0]	[0.467,0.5,0.033,0,0]	[0.461,0.5,0.039,0,0]
*x*_9_	[0,0,0.4,0.5,0.1]	[0,0,0.429,0.5,0.072]	[0,0,0.355,0.5,0.145]	[0,0,0.265,0.5,0.235]
*x*_10_	[0.5,0.5,0,0,0]	[0.556,0.444,0,0,0]	[0.556,0.444,0,0,0]	[0.5,0.5,0,0,0]
*x*_11_	[0.922,0.078,0,0,0]	[0.417,0.5,0.083,0,0]	[0.752,0.248,0,0,0]	[0.635,0.365,0,0,0]
*x*_12_	[0,0.245,0.5,0.255,0]	[0,0.48,0.5,0.02,0]	[0,0.395,0.5,0.105,0]	[0,0.235,0.5,0.265,0]
*x*_13_	[0,0.05,0.5,0.45,0]	[0,0.345,0.5,0.155,0]	[0.095,0.5,0.405,0,0]	[0.23,0.5,0.27,0,0]
*x*_14_	[0.556,0.444,0,0,0]	[0.337,0.5,0.163,0,0]	[0.393,0.5,0.107,0,0]	[0.44,0.5,0.06,0,0]
*x*_15_	[0,0.018,0.5,0.482,0]	[0,0.122,0.5,0.378,0]	[0,0.214,0.5,0.286,0]	[0,0.143,0.5,0.357,0]

Notes: [0.714,0.286,0,0,0] represents the connection number component between the index and the five evaluation grades, corresponding to vijk in (Eq.10)

**Fig 3:**
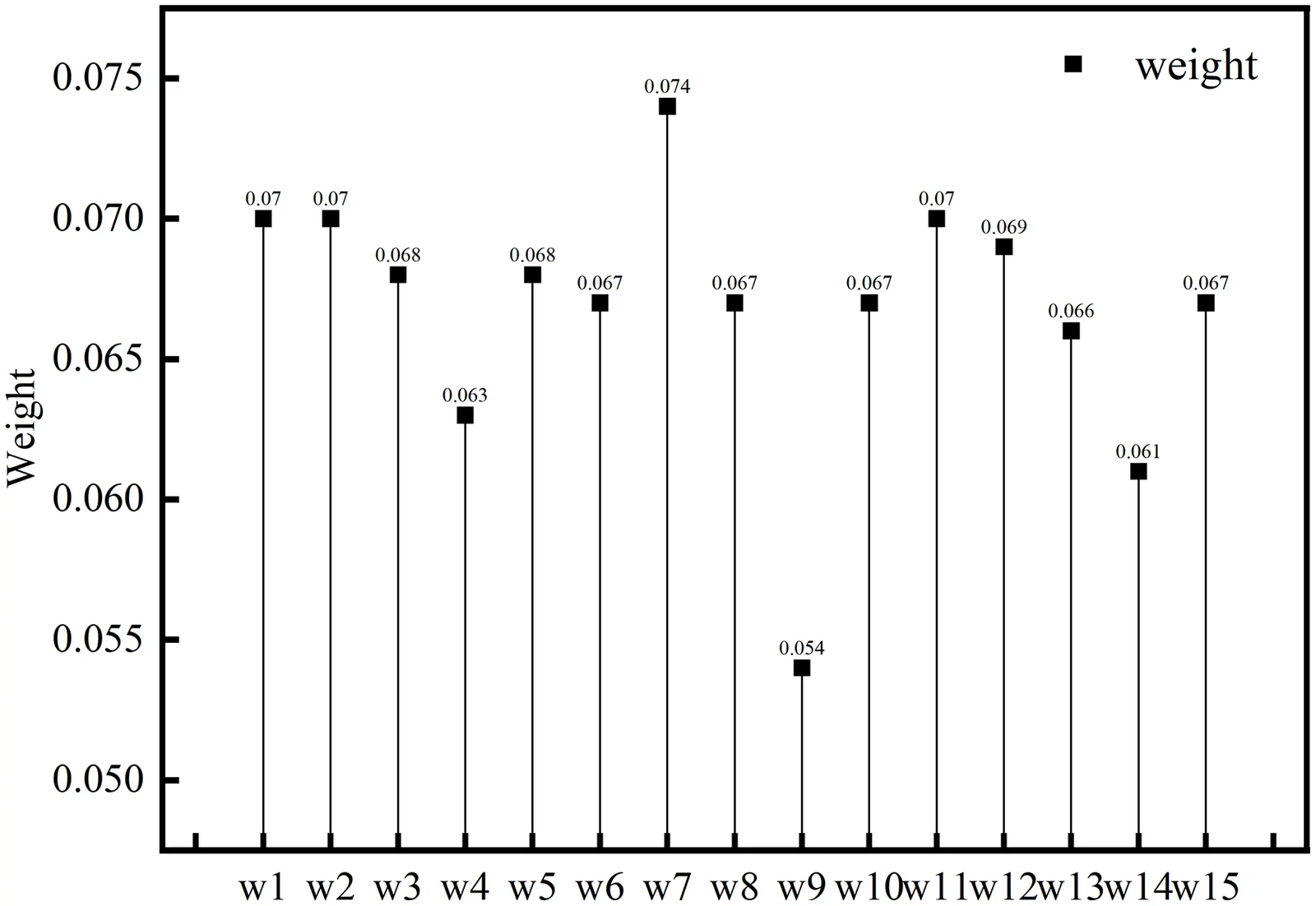
Weights of 15 evaluation indices.

**Fig 4:**
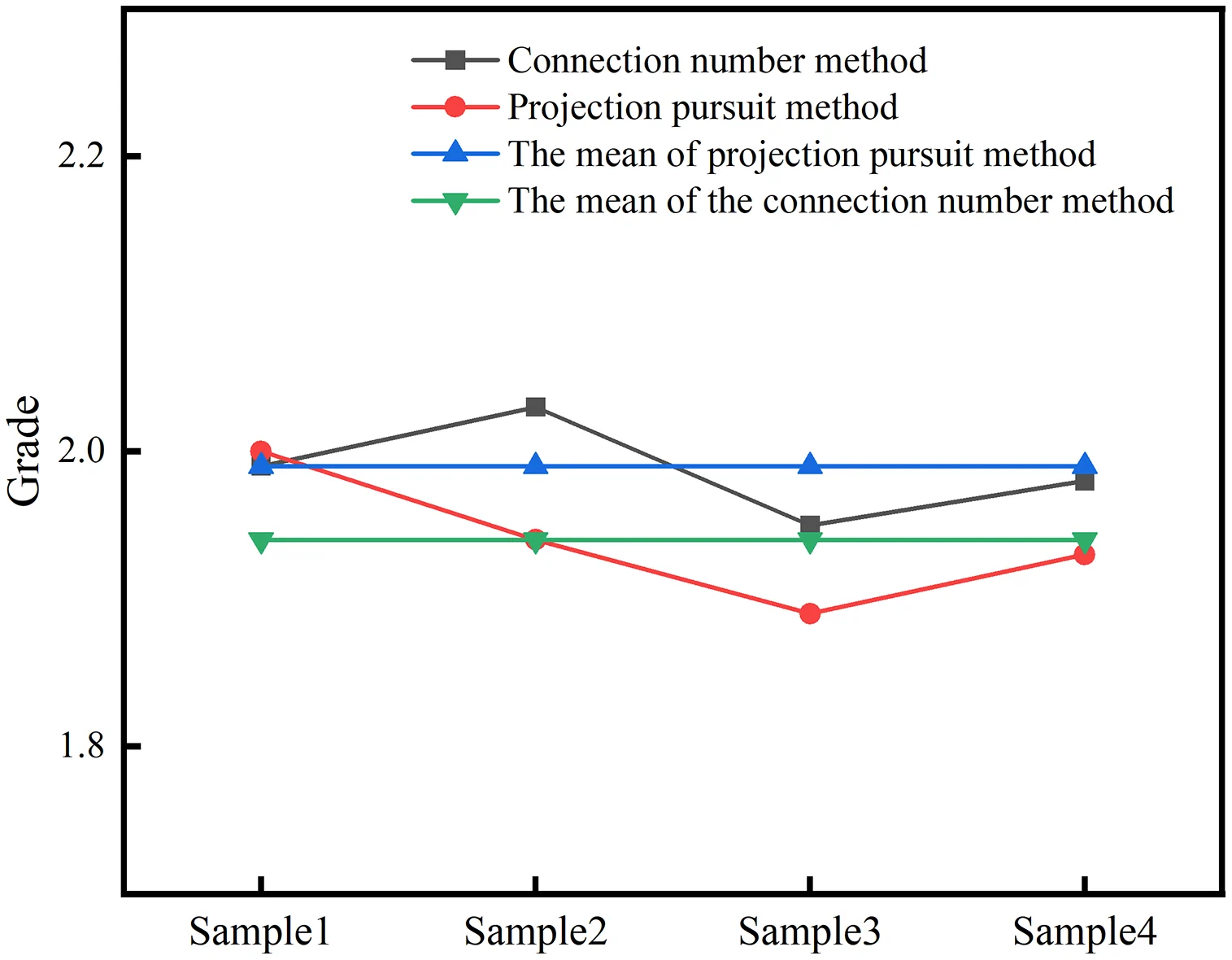
Groundwater quality evaluation grades calculated using two methods in Qiaocheng District, Bozhou City.

Index weights were extracted from the optimal projection direction derived in Section 3.1. Following absolute value transformation and normalization to avoid negative weight values, the final normalized weight vector was obtained as:

*w*=[0.07,0.07,0.068,0.063,0.068,0.067,0.074,0.067,0.054,0.067,0.07,0.069,0.066,0.061,0.067], shown in Fig 3:

The sample connection numbers for each groundwater monitoring site were calculated by substituting the single-index connection numbers and index weights into Eq.20. The grade eigenvalue method was adopted to convert these connection numbers into comprehensive groundwater quality grades for Qiaocheng District. The evaluation results derived from the set pair connection number model were compared with those obtained via the projection pursuit model, so as to verify the rationality of the projection pursuit framework and the reliability of the weight determination strategy based on the optimal projection direction. The evaluation results of the two methods are presented in Table 8 and Fig 4.

**Table 8:** Results of groundwater quality evaluation grade values calculated using the projection pursuit and connection number methods in Qiaocheng District, Bozhou City.

Samples	Connection number *u*_*i*_ of each evaluation sample					Results of this paper		Results of reference [30]	
	*a*	*b*_1_	*b*_2_	*b*_3_	*c*	Connection number method	Projection pursuit method	single-factor index method	Nemerow index method
1	0.430	0.278	0.170	0.116	0.005	1.99	2	4	clean
2	0.335	0.381	0.212	0.068	0.004	2.03	1.94	4	clean
3	0.386	0.363	0.174	0.069	0.008	1.95	1.89	4	clean
4	0.372	0.378	0.161	0.076	0.013	1.98	1.93	4	Lightly Polluted
mean	/	/	/	/	/	1.99	1.94	/	/

From the perspective of the connection number component characteristics, the opposition components *c* of the connection numbers for the four sets of groundwater monitoring samples in Qiaocheng District, Bozhou City, are all less than 0.1, with three sets of samples having opposition components below 0.01. Meanwhile, the similarity components of all samples are greater than 0.3, and the sum of the connection components for Grades I and II is greater than 0.6. This indicates that the overall groundwater quality in the study area is closer to the Class I or Class II standards and far from the Class V standard. This pattern is consistent with the conclusions derived from the comprehensive evaluation.

The evaluation grades obtained by the Set Pair Analysis (SPA) connection number method range from 1.95 to 2.03, while the comprehensive groundwater quality grades in Qiaocheng District, Bozhou City, calculated by the Projection Pursuit (PP) method range between 1.89 and 2.00. The evaluation grade values and their mean values from both methods are highly consistent, and the results corroborate each other.

### 3.3. Sensitivity analysis of index weights

**Fig 5:**
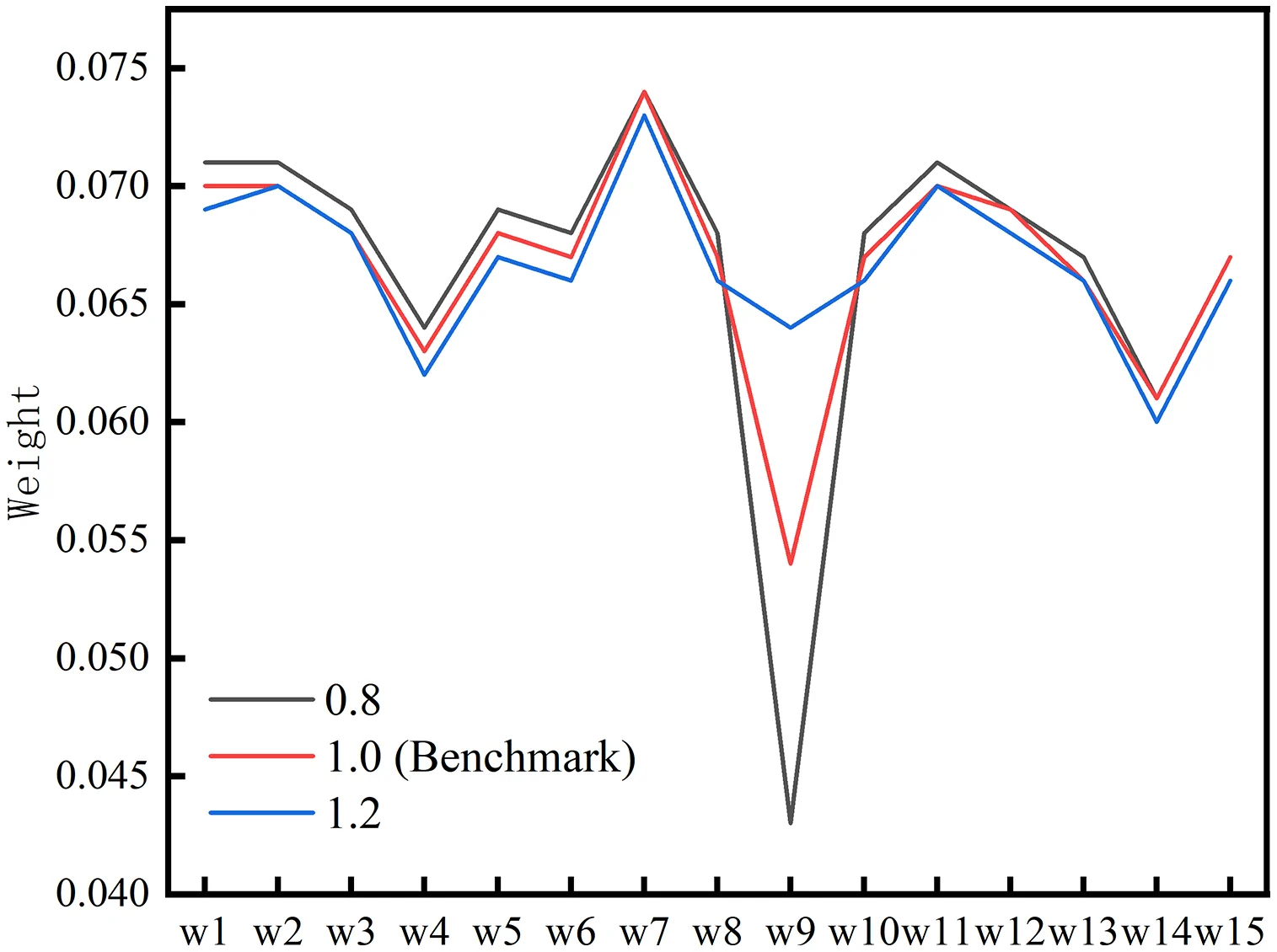
Sensitivity verification of weights under the Perturbation of Index *x*_9_.

When a scaling perturbation is applied to the *x*_9_ index, the weights determined based on the optimal projection direction of the PP method yield the following results (Table 9 and Fig 5):

**Table 9:** Sensitivity analysis results.

Disturbance Coefficient	0.8	1.0 (Benchmark)	1.2	Relative Change Rate (0.8 vs 1.0)	Relative Change Rate (1.2 vs 1.0)
*x*_1_	0.071	0.07	0.069	1.43%	−1.43%
*x*_2_	0.071	0.07	0.07	1.43%	0.00%
*x*_3_	0.069	0.068	0.068	1.47%	0.00%
*x*_4_	0.064	0.063	0.062	1.59%	−1.59%
*x*_5_	0.069	0.068	0.067	1.47%	−1.47%
*x*_6_	0.068	0.067	0.066	1.49%	−1.49%
*x*_7_	0.074	0.074	0.073	0.00%	−1.35%
*x*_8_	0.068	0.067	0.066	1.49%	−1.49%
***x***_**9**_ **(Target Disturbance Index)**	**0.043**	**0.054**	**0.064**	**−20.37%**	**18.52%**
*x*_10_	0.068	0.067	0.066	1.49%	−1.49%
*x*_11_	0.071	0.07	0.07	1.43%	0.00%
*x*_12_	0.069	0.069	0.068	0.00%	−1.45%
*x*_13_	0.067	0.066	0.066	1.52%	0.00%
*x*_14_	0.061	0.061	0.06	0.00%	−1.64%
*x*_15_	0.067	0.067	0.066	0.00%	−1.49%

(1) When the value of the *x*_9_ index is perturbed within a reasonable range, its corresponding weight exhibits a regular monotonic increase or decrease. As the scaling multiplier of the index value changes from 0.8 to 1.2, its weight value varies from 0.043 to 0.064. Using the weight corresponding to a multiplier of 1.0 as the baseline, the relative change rates are −20.37% and 18.52%, respectively. This demonstrates that the trend of weight changes is highly consistent with the physical driving mechanism of this index in the evolution of groundwater quality. The enhancement or attenuation of the index data can reasonably reflect the variations in its contribution to the comprehensive groundwater evaluation results.
(2) Apart from the *x*_9_ index, the relative change rates of the weights for the remaining 14 unperturbed indices are all within 2%. They only exhibit minor passive fluctuations, with no abnormal phenomena such as weight ranking disorder or logical reversal of contribution degrees.

### 3.4. Comparative verification based on multiple weighting method

**Fig 6:**
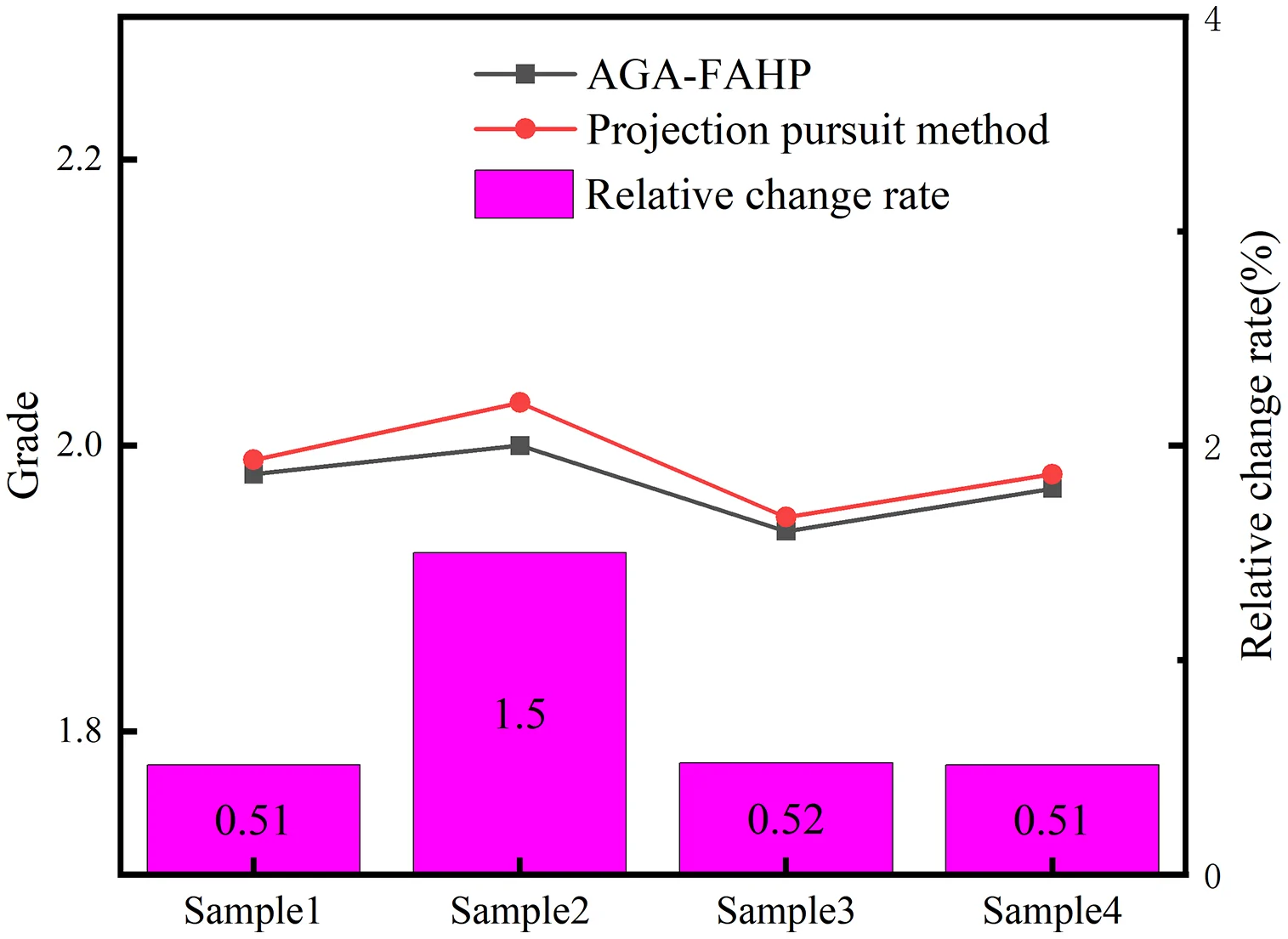
Independence verification of the weighting system.

By comparing the two sets of weight sequences, it is observed that their overall distributions are generally consistent, with no fundamental reversal in weight rankings. When the two sets of weights are respectively inputted into the connection number evaluation model described in Section 2.3.2, the evaluation results for the main urban area of Bozhou City are obtained as follows (Table 10 and Fig 6):

**Table 10:** Groundwater quality grades under different weighting methods in Qiaocheng District, Bozhou City.

Samples	Connection number *u*_*i*_ of each evaluation sample					Grades under different weighting methods		Relative change rate
	*a*	*b*_1_	*b*_2_	*b*_3_	*c*	AGA-FAHP	Projection pursuit method	
1	0.431	0.287	0.163	0.113	0.006	1.98	1.99	0.51%
2	0.346	0.381	0.203	0.066	0.004	2	2.03	1.50%
3	0.393	0.365	0.166	0.067	0.009	1.94	1.95	0.52%
4	0.379	0.375	0.153	0.077	0.015	1.97	1.98	0.51%
mean	/	/	/	/	/	1.97	1.94	/

As shown in the table, the matching consistency rate of the sample water quality grades is high. The mean evaluation grade values of the four samples are 1.97 and 1.94, respectively, and the relative change rates of the evaluation grades among the samples are all less than 2%. The evaluation results under the two sets of weights are highly consistent.

## 4. Discussion

### 4.1. Groundwater quality evaluation

#### 4.1.1. Fitting evaluation of the projection pursuit model.

The Projection Pursuit (PP) model demonstrates excellent fitting performance on the 150 standard samples. It achieves a high coefficient of determination (^2^ = 0.96), low RMSE (0.29) and MAE (0.25), and a classification accuracy of 100%. This indicates that the established projection objective function effectively captures the intrinsic correlation between the multidimensional water quality indices and the pre-determined groundwater quality grades. The outstanding fitting performance strongly validates the rationality of the model framework, optimization strategy, and sample calibration scheme adopted in this study, laying a solid theoretical foundation for the subsequent evaluation of groundwater quality at actual monitoring sites.

Despite the overall excellent fitting performance, the maximum prediction error among the samples is 0.5, indicating that there is still a half-grade deviation between the predicted grades and the true labels for a very small number of standard samples. This discrepancy may be attributed to the randomness of the data distribution generated by random sampling within the grade intervals, as well as minor fitting deviations for boundary samples during the Logistic fitting transformation process.

#### 4.1.2. Weighting mechanism of projection pursuit and the intrinsic causes of the low fluoride ion weight.

The weight of the 9th index, fluoride ion concentration, is relatively low at 0.054. This phenomenon can be comprehensively explained by combining the optimization mechanism of the Projection Pursuit (PP) model with the data distribution characteristics of the groundwater samples.

As a “grey-box” model, the core optimization objectives of the PP model are twofold: first, to maximize the standard deviation of the comprehensive projection values, thereby fully extracting the sample variation information embedded in high-dimensional indices to achieve optimal differentiation among evaluation results at different sampling sites; second, to ensure that the comprehensive projection values derived from the optimal projection direction possess strong explanatory power for the empirical regional groundwater quality grades. As a versatile optimization algorithm, the Accelerated Genetic Algorithm (AGA) features high computational efficiency and strong global adaptability. During the global optimization process, the model automatically assigns higher weights to indices with high inter-sample variation that can effectively differentiate sample differences, while assigning relatively lower weights to indices with small data fluctuations and uniform spatial distributions. Meanwhile, sensitivity tests on the index weights confirm that the evolutionary trajectory of the weights is highly positively correlated with the fluctuation amplitude of the index data. This endows the weight allocation with clear physical significance and validates its rationality.

According to GB/T 14848–2017, the standard thresholds for fluoride ions across different grades are relatively narrow. Consequently, the overall dispersion of the 150 sets of standard training sample data generated by random simulation is weak, with a relatively uniform spatial distribution. Therefore, when the Genetic Algorithm seeks the optimal projection direction through global optimization, it maximizes the extraction of overall variation information from multidimensional water quality indices, automatically reducing the weight of the fluoride ion concentration index. The groundwater source monitoring data from four sites in this study show that the fluoride concentrations at monitoring points 1, 2, 3, and 4 are 1.200 mg/L, 1.143 mg/L, 1.290 mg/L, and 1.470 mg/L, respectively, indicating weak dispersion. This finding is consistent with the conclusion of Li et al. [50], who applied the PP method for objective weighting in the groundwater quality evaluation of Yangquan City, China, and also found the fluoride ion concentration to have the lowest weight.

#### 4.1.3. Component characteristics of connection numbers and cross-validation of dual-model evaluation results.

As a “white-box” model for handling uncertainty, Set Pair Analysis (SPA) can further reveal the intrinsic distribution patterns of regional groundwater quality through the structural decomposition characteristics of the set pair connection number. In this study, the identity component of all four sets of groundwater monitoring samples is greater than 0.3. Meanwhile, the sum of the connection number components belonging to both Grade I and Grade II is greater than 0.6, and the contrary component is generally lower than 0.1. This set of component characteristics intuitively explains why the regional groundwater quality is overall closest to the Class I and Class II water quality standards, with virtually no sites tending toward the poor-quality Class V groundwater.

In the groundwater quality evaluation of the main urban area of Bozhou City, two models with entirely different mechanisms yielded highly consistent evaluation results. The evaluation results of the Projection Pursuit (PP) model ranged from 1.89 to 2, while the grade characteristic values of the Set Pair Connection Number model ranged from 1.95 to 2.03. The numerical values of the two models are highly convergent (with a grade value deviation of <0.1). This phenomenon of “reaching the same destination via different routes” strongly proves that the evaluation results do not stem from accidental errors of a specific algorithm, but truly reflect the objective laws of regional water quality. This significantly enhances the robustness and universality of the evaluation conclusions, demonstrating excellent adaptability to complex evaluation systems with multiple indices and high coupling in groundwater, and providing a reliable technical solution for the quantitative assessment of regional groundwater quality.

Traditional groundwater quality evaluation is highly susceptible to classification deviations for monitoring samples near critical thresholds, and it struggles to quantify the inherent fuzzy and random uncertainties within the evaluation system. The connection number model can decompose the correlation between monitoring samples and water quality standards of various grades into three characteristic components: similarity, difference, and opposition. By quantifying the membership tendency of samples to different water quality grades, this method explains the attribution mechanism of water quality grades at the data structure level. It can accurately characterize the gradual membership features of samples across multi-grade water quality standards, achieving a quantitative representation of the system’s fuzzy uncertainty. This effectively compensates for the defects of rigid deterministic classification boundaries and distorted classification of critical samples in traditional methods, demonstrating strong applicability and application advantages in the field of fuzzy comprehensive evaluation of groundwater quality.

#### 4.1.4. Analysis of the limitations of traditional evaluation methods and the advantages of the proposed models.

Regarding the groundwater quality issues in the main urban area of Bozhou City, previous study [30] have provided crucial baseline evaluation data and references. These studies indicate that the fluoride concentration in the regional groundwater exceeds the Class III water quality limit of 1.00 mg/L, primarily due to geological factors such as the paleo-depositional environment. Subsequent research [51] has further elucidated the hydrogeochemical mechanisms, identifying fluorite as the core source of fluoride. Moreover, the precipitation of calcite and cation exchange within the aquifer continuously disrupt the dissolution equilibrium of fluorite, clarifying the formation mechanism of primary high-fluoride groundwater in this area.

However, in the water quality evaluation of such primary high-fluoride areas, the traditional single-factor evaluation method often designates the entire region as Class IV water solely based on fluoride exceedance. This approach has inherent limitations: it tends to amplify the negative impact of a single geological exceedance index while ignoring the comprehensive improvement of other water quality parameters, which may lead to biased overall water quality assessments for the region. Meanwhile, this method struggles to distinguish the micro-level quality differences among water bodies within the same grade, limiting its applicability.

In contrast, the calculation results of the Nemerow Index method are highly consistent with the evaluation outcomes of the two models proposed in this study (Projection Pursuit and Set Pair Connection Number), which corroborates the reliability of our evaluation system. Nevertheless, the Nemerow Index method only outputs a comprehensive pollution index. It relies on empirical thresholds to classify pollution levels and cannot directly output the standardized water quality grades (Classes I–V) defined by national standards, leaving room for improvement in terms of result intuitiveness.

To address these shortcomings, the Projection Pursuit (PP) model constructed in this study is based on a “simulation training-projection pursuit” data-driven mechanism. It focuses on extracting the intrinsic structural features and dimensionality reduction mapping of multidimensional data, achieving objective weighting. The robustness of this weighting system has been verified through sensitivity and independence analyses. On the other hand, the connection number model focuses on quantifying the uncertain membership relationships between the evaluation objects and the grades, effectively overcoming the issue of fuzzy critical boundaries in index methods and achieving continuous quantification of water quality grades. More importantly, although these two models possess entirely different mathematical mechanisms, their evaluation results exhibit high consistency. This cross-validation based on different mechanisms strongly rules out accidental errors that might be caused by a single algorithm, proving that the evaluation results genuinely reflect the objective laws of regional water quality, thereby significantly enhancing the robustness and universality of the evaluation conclusions.

Overall, the general groundwater quality grade in Qiaocheng District, Bozhou City, is Grade 2, corresponding to Class II groundwater. According to the Quality Standard for Ground Water (GB/T 14848–2017), the groundwater in Qiaocheng District can serve as a centralized source for domestic drinking water. However, according to the Investigation Report on Land Subsidence in Bozhou City, the water level of the middle-layer groundwater in Qiaocheng District has shown a slow declining trend since 2003, with an average annual drop of approximately 0.22 meters, mainly due to excessive extraction of middle-layer groundwater in the urban area. Therefore, scientific and rational management of groundwater extraction activities is necessary during socio-economic development. In 2019, the South-to-North Water Diversion Project introduced Yangtze River water into the urban area of Bozhou City, bringing the entire city into the project’s water supply coverage. This has created favorable conditions for substituting local groundwater sources and reducing groundwater extraction volumes.

### 4.2. Rationality analysis of projection pursuit weighting strategy

The weight of x9 exhibits a monotonic variation in response to index perturbation. This changing trend aligns highly with the physical driving mechanism of the index in the evolution of groundwater quality. The enhancement or attenuation of the index data can reasonably reflect the variation in its contribution to the comprehensive groundwater evaluation results. This indicates that the Projection Pursuit (PP) weighting possesses a reasonable physical connotation, and the weighting method is scientifically effective.

The remaining indices show only minor passive fluctuations, and the weight structure remains stable without disorder. This demonstrates that the optimal projection direction of the PP model is not merely a mathematically random optimal solution. Instead, the weight allocation conforms to physical meanings and exhibits strong robustness.

Based on the comprehensive results of the perturbation tests, the statistical robustness and physical interpretability of the weights in this study have been verified from the perspective of sensitivity, thereby supporting the scientific validity of the evaluation system.

### 4.3. Independent verification based on multiple weighting methods

To conduct a comparative experiment, the AGA-FAHP [49] objective weighting method was introduced in this study. The results indicate that even when an independent objective weighting system was applied, the comprehensive evaluation grades, spatial evolution patterns, and dominant controlling indices of the regional groundwater quality remained essentially unchanged. This demonstrates that the evaluation conclusions based on the original Projection Pursuit-Connection Number model are not accidental outcomes caused by the endogenous parameters of a specific algorithm. Furthermore, this effectively eliminates the credibility defects that may arise from process cross-intersection between the weighting and evaluation models.

Meanwhile, the high consistency of the evaluation results under the two sets of weights further corroborates that the index weights derived from the optimal projection direction of the Projection Pursuit model possess clear physical significance and statistical stability. Combined with the univariate sensitivity analysis results discussed previously, the scientific validity and robustness of the evaluation system in this study are comprehensively demonstrated from two dimensions: methodological independence and parameter sensitivity.

### 4.4. Limitations and future research perspectives

Limited number of monitoring samples: This study relies solely on water quality data from four fixed monitoring sites for evaluation. The low spatial density of the samples makes it difficult to fully and precisely characterize the small-scale spatial heterogeneity of groundwater quality in the study area, thereby limiting the ability to identify localized micro-pollution patches.

Recommendation for dynamic monitoring: Conducting long-term, seasonal, and multi-temporal dynamic monitoring is recommended. This will help explore the impacts of hydrological variations between wet and dry seasons on groundwater mineralization, ion migration, and water quality grade fluctuations, ultimately achieving a dynamic water quality assessment.

Applicability limited to static status evaluation: The coupled evaluation model constructed in this study currently only achieves a static grading evaluation of the present groundwater quality status. A time-series prediction module has not yet been established, making it impossible to predict future water quality evolution trends. Thus, the early warning capability of the model needs to be further expanded.

## 5. Conclusions

To address the inherent limitations in groundwater quality evaluation, such as the strong subjectivity in weighting, the misclassification of critical samples, and the restricted perspective of single-model evaluations, this study constructs two evaluation models with completely independent mathematical mechanisms: Projection Pursuit (PP) and connection number These two models are employed to achieve cross-validation of the evaluation results. The AGA is introduced to optimize the projection direction, thereby realizing the objective weighting of multidimensional water quality indices. Furthermore, by decomposing the water quality grades into similarity, difference, and opposition components via the connection number model, the fuzzy uncertainty within the evaluation system is effectively quantified. The main conclusions are as follows:

The comprehensive groundwater quality in Qiaocheng District, Bozhou City, is classified as Grade Ⅱ, with a continuous grade characteristic value ranging from 1.95 to 2.03. The overall water quality is excellent, making it suitable as a centralized source of domestic drinking water. Controlled by the primary sedimentary hydrogeochemical background, fluoride in the area is slightly above the standard locally, but this does not alter the overall excellent water quality pattern. The annual average decline in the middle-layer groundwater level is 0.22 m, indicating continuously intensifying groundwater extraction pressure. It is recommended to rely on the South-to-North Water Diversion Project (Yangtze River replenishment) to rationally reduce the scale of groundwater extraction, ensuring the balanced stability of the regional groundwater system.

The index weights derived from the Projection Pursuit model possess clear physical connotations. The relatively low weight of the fluoride index stems from its uniform spatial distribution and weak overall sample variation, which aligns with the optimization logic of Projection Pursuit to maximize the extraction of sample variation information. Dual verification through independent weighting comparative tests and index perturbation sensitivity analysis demonstrates that this objective weighting system exhibits excellent scientific validity and robustness.

The two models with completely distinct mathematical mechanisms achieved highly consistent calculation results in the groundwater evaluation of the study area. This proves that the evaluation conclusions are not disturbed by a single algorithm and can objectively reflect the inherent laws of regional groundwater quality. The coupled evaluation framework constructed in this paper is highly adaptable to complex evaluation systems characterized by multiple indices and strong coupling in groundwater. It significantly enhances the credibility and universality of the evaluation results, providing reliable technical support for the quantitative evaluation of groundwater quality and water resource management in similar areas of the Northern Anhui Plain.

## Supporting information

S1 Table Comparison of empirical values and projection pursuit model calculation values for groundwater quality grades. (DOCX)
