# Supplementary material for: Construction and application of connection number and projection pursuit models for groundwater quality evaluation: A case study of the main urban area of Bozhou City
Source: PLoS One. 2026 Aug 27;21(8):e0356475. doi: 10.1371/journal.pone.0356475 (PMC13521414; doi:10.1371/journal.pone.0356475)
Supplement: Supplementary file 1 [file pone.0356475.s001.docx]

**S1 Table Comparison of empirical values and projection pursuit model calculation values for groundwater quality grades**

| **Samples** | **Projection value** | **Grade** | |
| --- | --- | --- | --- |
|  |  | **Empirical values** | **Projection pursuit model calculation values** |
| 1 | 0.109 | 1 | 1.45 |
| 2 | 0.100 | 1 | 1.43 |
| 3 | 0.085 | 1 | 1.39 |
| 4 | 0.093 | 1 | 1.41 |
| 5 | 0.121 | 1 | 1.47 |
| 6 | 0.115 | 1 | 1.46 |
| 7 | 0.101 | 1 | 1.43 |
| 8 | 0.073 | 1 | 1.37 |
| 9 | 0.062 | 1 | 1.34 |
| 10 | 0.065 | 1 | 1.35 |
| 11 | 0.083 | 1 | 1.39 |
| 12 | 0.101 | 1 | 1.43 |
| 13 | 0.032 | 1 | 1.28 |
| 14 | 0.116 | 1 | 1.46 |
| 15 | 0.101 | 1 | 1.43 |
| 16 | 0.107 | 1 | 1.44 |
| 17 | 0.078 | 1 | 1.38 |
| 18 | 0.102 | 1 | 1.43 |
| 19 | 0.097 | 1 | 1.42 |
| 20 | 0.100 | 1 | 1.43 |
| 21 | 0.110 | 1 | 1.45 |
| 22 | 0.114 | 1 | 1.46 |
| 23 | 0.039 | 1 | 1.30 |
| 24 | 0.128 | 1 | 1.49 |
| 25 | 0.093 | 1 | 1.41 |
| 26 | 0.090 | 1 | 1.40 |
| 27 | 0.055 | 1 | 1.33 |
| 28 | 0.087 | 1 | 1.40 |
| 29 | 0.098 | 1 | 1.42 |
| 30 | 0.089 | 1 | 1.40 |
| 31 | 0.274 | 2 | 1.84 |
| 32 | 0.294 | 2 | 1.89 |
| 33 | 0.250 | 2 | 1.78 |
| 34 | 0.290 | 2 | 1.88 |
| 35 | 0.290 | 2 | 1.88 |
| 36 | 0.286 | 2 | 1.87 |
| 37 | 0.224 | 2 | 1.71 |
| 38 | 0.277 | 2 | 1.85 |
| 39 | 0.281 | 2 | 1.86 |
| 40 | 0.274 | 2 | 1.84 |
| 41 | 0.302 | 2 | 1.91 |
| 42 | 0.257 | 2 | 1.79 |
| 43 | 0.268 | 2 | 1.82 |
| 44 | 0.274 | 2 | 1.84 |
| 45 | 0.281 | 2 | 1.86 |
| 46 | 0.309 | 2 | 1.93 |
| 47 | 0.278 | 2 | 1.85 |
| 48 | 0.251 | 2 | 1.78 |
| 49 | 0.302 | 2 | 1.91 |
| 50 | 0.250 | 2 | 1.78 |
| 51 | 0.273 | 2 | 1.84 |
| 52 | 0.297 | 2 | 1.90 |
| 53 | 0.274 | 2 | 1.84 |
| 54 | 0.267 | 2 | 1.82 |
| 55 | 0.274 | 2 | 1.84 |
| 56 | 0.311 | 2 | 1.93 |
| 57 | 0.272 | 2 | 1.83 |
| 58 | 0.288 | 2 | 1.87 |
| 59 | 0.276 | 2 | 1.84 |
| 60 | 0.265 | 2 | 1.82 |
| 61 | 0.557 | 3 | 2.59 |
| 62 | 0.610 | 3 | 2.73 |
| 63 | 0.635 | 3 | 2.79 |
| 64 | 0.593 | 3 | 2.68 |
| 65 | 0.553 | 3 | 2.57 |
| 66 | 0.614 | 3 | 2.74 |
| 67 | 0.547 | 3 | 2.56 |
| 68 | 0.563 | 3 | 2.60 |
| 69 | 0.549 | 3 | 2.57 |
| 70 | 0.552 | 3 | 2.57 |
| 71 | 0.591 | 3 | 2.68 |
| 72 | 0.581 | 3 | 2.65 |
| 73 | 0.592 | 3 | 2.68 |
| 74 | 0.615 | 3 | 2.74 |
| 75 | 0.552 | 3 | 2.57 |
| 76 | 0.634 | 3 | 2.79 |
| 77 | 0.544 | 3 | 2.55 |
| 78 | 0.586 | 3 | 2.66 |
| 79 | 0.588 | 3 | 2.67 |
| 80 | 0.582 | 3 | 2.65 |
| 81 | 0.628 | 3 | 2.78 |
| 82 | 0.615 | 3 | 2.74 |
| 83 | 0.644 | 3 | 2.82 |
| 84 | 0.588 | 3 | 2.67 |
| 85 | 0.525 | 3 | 2.50 |
| 86 | 0.628 | 3 | 2.78 |
| 87 | 0.562 | 3 | 2.60 |
| 88 | 0.608 | 3 | 2.72 |
| 89 | 0.629 | 3 | 2.78 |
| 90 | 0.553 | 3 | 2.57 |
| 91 | 1.234 | 4 | 4.11 |
| 92 | 1.464 | 4 | 4.42 |
| 93 | 1.336 | 4 | 4.26 |
| 94 | 1.413 | 4 | 4.36 |
| 95 | 1.321 | 4 | 4.24 |
| 96 | 1.367 | 4 | 4.30 |
| 97 | 1.393 | 4 | 4.34 |
| 98 | 1.226 | 4 | 4.10 |
| 99 | 1.423 | 4 | 4.37 |
| 100 | 1.250 | 4 | 4.14 |
| 101 | 1.397 | 4 | 4.34 |
| 102 | 1.431 | 4 | 4.38 |
| 103 | 1.420 | 4 | 4.37 |
| 104 | 1.451 | 4 | 4.40 |
| 105 | 1.455 | 4 | 4.41 |
| 106 | 1.457 | 4 | 4.41 |
| 107 | 1.472 | 4 | 4.43 |
| 108 | 1.340 | 4 | 4.27 |
| 109 | 1.323 | 4 | 4.24 |
| 110 | 1.401 | 4 | 4.35 |
| 111 | 1.405 | 4 | 4.35 |
| 112 | 1.430 | 4 | 4.38 |
| 113 | 1.497 | 4 | 4.45 |
| 114 | 1.308 | 4 | 4.22 |
| 115 | 1.312 | 4 | 4.23 |
| 116 | 1.501 | 4 | 4.46 |
| 117 | 1.227 | 4 | 4.10 |
| 118 | 1.415 | 4 | 4.36 |
| 119 | 1.399 | 4 | 4.34 |
| 120 | 1.488 | 4 | 4.45 |
| 121 | 2.890 | 5 | 4.97 |
| 122 | 2.913 | 5 | 4.97 |
| 123 | 3.024 | 5 | 4.98 |
| 124 | 2.716 | 5 | 4.96 |
| 125 | 2.907 | 5 | 4.97 |
| 126 | 2.844 | 5 | 4.97 |
| 127 | 2.995 | 5 | 4.98 |
| 128 | 2.890 | 5 | 4.97 |
| 129 | 2.480 | 5 | 4.93 |
| 130 | 2.930 | 5 | 4.97 |
| 131 | 3.061 | 5 | 4.98 |
| 132 | 2.993 | 5 | 4.98 |
| 133 | 2.826 | 5 | 4.97 |
| 134 | 3.154 | 5 | 4.98 |
| 135 | 2.751 | 5 | 4.96 |
| 136 | 2.754 | 5 | 4.96 |
| 137 | 2.739 | 5 | 4.96 |
| 138 | 3.136 | 5 | 4.98 |
| 139 | 3.033 | 5 | 4.98 |
| 140 | 3.081 | 5 | 4.98 |
| 141 | 2.800 | 5 | 4.96 |
| 142 | 2.907 | 5 | 4.97 |
| 143 | 2.966 | 5 | 4.97 |
| 144 | 2.863 | 5 | 4.97 |
| 145 | 2.691 | 5 | 4.95 |
| 146 | 3.181 | 5 | 4.98 |
| 147 | 3.017 | 5 | 4.98 |
| 148 | 2.957 | 5 | 4.97 |
| 149 | 2.839 | 5 | 4.97 |
| 150 | 2.740 | 5 | 4.96 |
